# Supplementary material for: Plasma proteome perturbation for CMV DNAemia in kidney transplantation
Source: PLoS One. 2023 May 19;18(5):e0285870. doi: 10.1371/journal.pone.0285870 (PMC10198483; doi:10.1371/journal.pone.0285870)
Supplement: S1 Fig — (DOCX) [file pone.0285870.s001.docx]

**Supplemental Figures**

**S1 Fig.** A bubble plot demonstrating relative fold increase and decrease and statistical significance of the proteins that were either increased (n=6) or decreased (n=11) in pre-CMV infection samples in CMV DNAemia positive patients compared to matching CMV DNAemia negative cohort.
