# Supplementary material for: Plasma proteome perturbation for CMV DNAemia in kidney transplantation
Source: PLoS One. 2023 May 19;18(5):e0285870. doi: 10.1371/journal.pone.0285870 (PMC10198483; doi:10.1371/journal.pone.0285870)
Supplement: S2 Fig — In between transcriptome and proteome data, the average correlation for the most significant targets at the time of CMV DNAemia was 0.11, with the highest observed correlation between proteome and transcriptome seen in immunoglobulin heavy constant gamma 1 (IGHG1) (Pearson, r = 0.44) (2A). There are six unique proteins when observing the differentially expressed CMV DNAemia positive signature between transcriptome and proteome. Among the six genes and proteins existing in the CMV signature, Orosomucoid 1 (ORM1) holds a positive correlation (Pearson, r = 0.17) (2B). (DOCX) [file pone.0285870.s002.docx]

**S2 Fig. Correlation analysis of transcriptome and proteome data.** In between transcriptome and proteome data, the average correlation for the most significant targets at the time of CMV DNAemia was 0.11, with the highest observed correlation between proteome and transcriptome seen in immunoglobulin heavy constant gamma 1 (IGHG1) (Pearson, r=0.44) (**A**). There are six unique proteins when observing the differentially expressed CMV DNAemia positive signature between transcriptome and proteome. Among the six genes and proteins existing in the CMV signature, Orosomucoid 1 (ORM1) holds a positive correlation (Pearson, r=0.17) (**B**).
